# General practice specialty decision making: a system-level Australian qualitative study

**DOI:** 10.3399/BJGPO.2024.0218

**Published:** 2025-07-30

**Authors:** Faith R Yong, Priya Martin, Katharine A Wallis, Jordan Fox, Sneha Kirubakaran, Riitta L Partanen, Srinivas Kondalsamy-Chennakesavan, Matthew R McGrail

**Affiliations:** 1 Rural Clinical School, Faculty of Medicine, The University of Queensland, Queensland, Australia; 2 School of Health and Medical Sciences, University of Southern Queensland, Queensland, Australia; 3 General Practice Clinical Unit, Faculty of Medicine, The University of Queensland, Queensland, Australia

**Keywords:** Qualitative research, Workforce, Medical education, General practice, Primary healthcare, General practitioners

## Abstract

**Background:**

Ensuring sufficient supply of GPs is critical for servicing increasing healthcare demands. Heightened by pandemic conditions, chronic shortages of GPs persist globally. While many factors reinforcing the choice of general practice as a specialty are known, system-level understanding of factors that influence GPs’ career decision making during their medical training requires investigation.

**Aim:**

To explore the reasons for specialty choice through career selection narratives of recently registered Australian GPs, using a system-level perspective.

**Design & setting:**

Semi-structured interviews were selected for in-depth explorations of the rationale for choosing general practice as a specialty. Within Australia, medical specialty training choices are typically made after both university medical education and mandatory 1–2 year pre-vocational (hospital-based) training have been completed.

**Method:**

Interviews were conducted online with GPs who had completed all training in the last 10 years. De-identified and verified transcripts underwent participant checking. Deductive framework analysis, using career counselling constructs, and inductive thematic analysis were performed.

**Results:**

There were 25 participants. Career counselling constructs provided system-level understanding of GP specialty decision-making processes. Many participants highlighted that there were large gaps in information about a GP career throughout medical training. Overcoming negative medical narratives about general practice was necessary for most in choosing a GP career. However, positive experiences with GP communities or work gave insights into the broad flexibility of their person–specialty fit with general practice.

**Conclusion:**

GP work experiences and personal GP connections could counteract prominent negative narratives about GP careers. However, lack of systemic and regular exposure to GPs throughout medical training is a critical barrier that should be addressed through sustained policy and professional interventions.

## How this fits in

Both attractive factors about the general practice specialty and negative medical narratives within the profession about GP careers are well known. However, there is a lack of understanding about how these factors affect medical students and postgraduate doctors within medical training at a system level. This study provides an explanation of multiple, overlapping reasons that can result in a choice of general practice as a career, which includes contrasting the working conditions, medical preferences and autonomy associated with hospital (non-GP) and GP specialties. It seems a lack of systemic exposure to general practice work in Australia results in insufficient general practice experiences and connections to counteract widespread medical stigmatisation and context differences in GP careers, contributing to lessening uptake of GP training and career withdrawal.

## Introduction

A strong primary care workforce with sufficient doctors practising general medicine supports both system efficiencies and improved health outcomes.^1–3^ However, a global trend continues with more doctors specialising in narrow fields focused on targeted populations or body systems, rather than general practice (or family medicine).^4–6^ Any global successes of stabilising or increasing the supply of GPs have been counteracted by greater population health needs largely owing to increased life expectancy. More people are living with chronic disease and increased mental health needs, placing more stress on the primary care workforce.^7,8^ Moreover, the COVID-19 pandemic placed a disproportionate toll on GPs, leaving the workforce increasingly stressed, overworked and looking to depart the sector.^9^ The current ‘crisis’ in general practice of insufficient GP workforce numbers remains a ’wicked’ problem^10,11^ and a renewed understanding of what underpins and sustains the choice of general practice as a specialty is needed.^12,13^

Ideally, a sufficient supply of GPs would rely upon specialty choices aligning with the population’s healthcare needs.^14^ However, the choice of medical specialty is a multifactorial process^15–17^ predominantly centred on the individual doctor’s preferences.^18^ Most seek first-hand information about potential career choices, then test their ‘fit’ against both professional and non-professional personal priorities. This includes a range of intrinsic factors such as self-assessment of their skills, desire for prestige, or relative financial reward; and extrinsic factors such as work culture, hours of work, or training structure.^19^ Given the critical role of specialty distribution to the health system, many studies have specifically focused on factors relating to choosing general practice as a specialty.^4,20,21^ Common factors associated with general practice that are considered attractive include opportunities to practice continuity of care, variety of work, autonomy, control of work hours, lifestyle, and perceived work–life balance. However, deterrent factors include comparatively lower income to some specialties, limited opportunities for career advancement, preference for working within a team-based care environment rather than solo consulting, and its perceived lower professional status compared to other medical specialties.^22^

Australia, like many countries globally, continues to experience both location and profession distribution issues in its medical workforce.^23,24^ However, little system-level evidence regarding the decision to become a GP has been gathered across medical training stages in Australia.

This study is part one of an Australian two-phase investigation exploring areas where stronger integration of all stages of the GP education and training pipeline across the health system is needed. It aims to explore the rationale for specialty choice through career selection narratives of recently registered Australian GPs, using a system-level perspective. Phase two explores possible system integration solutions from GP perspectives and is reported elsewhere.

## Method

Semi-structured interviews were conducted to provide insights into GP career decision making, according to a constructivist approach.^25^ Qualitative reporting guidelines were followed.^26^ The research team was interprofessional to enhance the depth of insights obtained.

### Setting

Australian medical school training begins with a mix of school-leaver and postgraduate-entry medical schools, providing a total university training period of 5–7 years. All medical graduates then complete pre-vocational training, comprising 1–2 years internship plus an average 1–3 (or more) years working in varying hospital departments, before they finalise their specialty choices. Clinical training experiences in general practice settings of 6–12 weeks (up to 6–12 months in some programmes) are common for most medical students. However, the vast majority of pre-vocational training is conducted in hospital-based departments.

### Participant recruitment

Eligible participants were doctors who had completed GP registrar training to become independent GPs since 2014. In Australia, this equates to fellowship of either the Royal Australian College of General Practice (FRACGP) or the Australian College of Rural and Remote Medicine (FACRRM). Potential participants were contacted through: professional networks of research team members; University of Queensland GP educators and alumni lists; and recruitment notices in closed GP social media groups (for example, RACGP Queensland New Fellows Facebook [Meta] group). Notices and reminders included study aims, contact details, and links to the online participant information and consent form. Participating GPs were given a $150 (AUD) voucher as per RACGP guidelines in acknowledgement of their time spent in interviews. Recruitment continued until data saturation based on the interview questions was deemed to be reached with team consensus.

### Data collection

Online consent forms were followed by a demographic survey, availability for interviews, and contact information. The interview guide^15^ was updated for this study by MM, TG, PM, and FY; piloted with two GPs (including KW); then modified for clarity, brevity, and relevance to GPs (Supplementary Box 1). Questions were designed to explore decision making and GP work exposure in different stages of medical training, including medical school (that is, medical education) and postgraduate doctor (specifically, pre-vocational) training.

Individual, semi-structured interviews were conducted by experienced qualitative researchers, FY and TG (acknowledged), using Zoom Video Communications or Microsoft Teams. Interviews were recorded, transcribed verbatim by a third-party transcription company, then verified and de-identified using participant codes. Transcripts were provided to the respective participant for checking, and were deemed to have been acceptable to the participant if there was no response to the contrary within 14 days.

### Analysis

Participant demographics were tabulated. Framework method analysis^27^ of transcripts was undertaken by experienced qualitative researchers (FY and PM) in an iterative manner. After transcript familiarisation, it was determined that a theoretical framework would be helpful to explore medical pathway decision making from participants’ non-chronological career narratives. Xu’s dual-process theory of career decision-making (DTC) framework from career counselling^28^ was chosen for broad theoretical relevance, ease of application to medical pathways, and the explanatory nature of its constructs. The DTC framework also has an associated career decision-making process model of four cyclical macro stages. Stage 1 consists of broad exploration, stage 2 of ‘reducing confusion and ambiguity’, stage 3 of ‘implementing the choice’, and stage 4 relates to ‘re-evaluating the choice’.^29^

To understand participant narratives and the implications for GP pathways, the DTC framework was combined with an educational learning construct, ‘threshold concepts’ (that is, irreversible knowledge gain that decreased career confusion), for GP pathways.^30^ In other words, pivotal knowledge about GP careers that assisted and clarified decision making for GPs was coded as threshold concepts. Data were coded deductively according to these constructs to ensure that GPs’ career decision-making processes would be highlighted through the framework analysis process. This was an intermediary step to deconstruct career narratives in a format that would enable themes for GP career pathways across training stages. The framework constructs in regards to GP pathways were summarised briefly and illustrated with quotes (Table 1).

**Table 1.** Illustrative GP quotes relevant to Xu’s dual-process theory of career decision-making (DTC) framework constructs

Framework construct	Definition	Quote
*DTC framework* ^28^		
(1) Anchor choices	Intermediate choices that provide career direction, e.g. medical versus surgical, preferencing specialties or not, case variety, location, or community	*“I did a rotation in PGPPP* [Prevocational General Practice Placements Program] *in my second year out, which was a very positive experience. I really enjoyed that. I think that that’s a significant part of why I decided to go into general practice.”* (GP5)
(2) Agentic adjustment	Adjustment of initial career choice based on feedback, using critical self-reflection and career adaptability (that is, concerns about the future, curiosity in exploration, internal sense of control, coping confidence)	*“[…] a few of my colleagues are leaving general practice now and it’s because they’re burnt out from always bending over backwards trying to accommodate things, so I think you do have to find that sweet point between flexibility and where your boundaries have to be firmer. But if you find that then general practice is great.”* (GP6)
(3) Strategic planning	Non-linear, intentional style of career design with holistic, long-term oriented goals by the individual	*“[…] but all the time – if someone has started* obstetrics *training and then halfway through they said, ‘You know what, too many night shifts. I want to have a family. My partner earns enough money. I don’t need to work as a surgeon and, you know what, it’s too hard on my – I want to have kids and it’s not good for my training program. I’m going to leave. I’m going to become a GP.’” (GP9)*
(4) Managing ambiguity	How the individual self-manages thoughts about their career choice which they are not able to confirm or disconfirm before trying it out personally	*“[…] for that longer length of placement, it was definitely, ‘Yes, if general practice is your path, great. Follow it. If general practice really isn’t your path, great. Follow that.’ The others kind of still figure their own way out.”* (GP13)
(4a) Satisficing	Settling for a choice as ‘good enough’ by approaching and making attempts to understand any ambiguity, and acknowledging any ambiguity is acceptable	*“I think the number one challenge is – I mean for me it probably didn’t – I guess there was still that kind of lack of ‘I don’t really know what it is to be a GP,’ because you don’t have that exposure. I think that what that was still a challenge because I didn’t – I hadn’t had that kind of personal experience.”* (GP1)
(4b) Maximising	Approaching ambiguity by exhausting decision-making resources and efforts, and denying any ambiguity can exist	*“I narrowed it down till I was choosing between general practice and paediatrics, and had a whole lot of trouble making up my mind. I did a year at the children’s hospital and was on the paediatrics training pathway without actually having committed to doing that yet. Just thinking, well, I would have a year of that under my belt if I do decide to do that. Ended up applying for both, getting offers for both and taking it down to the wire in terms of the decision making.”* (GP5)
(4c) Ambiguity avoidance	Passive avoidance of ambiguity by denying its existence, since ambiguity is unacceptable; anxiety is a characteristic of this state	*“[…] I just defaulted to physician training because I didn’t want to do surgery, I didn’t want to do anaesthetics, I didn’t want to do radiology or any of the other super-niche things, so it just seemed like physician training was the default decision. So, I did that and then I just realised the job was crap and then I’m like, ‘Oh wait, there’s GP as an option.’ It almost came as a, not afterthought, but yeah, like a second line consideration for me.”* (GP10)
(4d) Calling and hope	Avoids ambiguity by not particularly doing anything to understand it, but points instead to a ‘higher calling’ to the career choice; accepts that such ambiguity exists. Requires emotionally empowered experiences and intuitive thinking to resolve ambiguity instead, and deliberate evaluation is unlikely to help	*“I didn’t even know what the pay was until I entered it. I hadn’t looked at the advertised – I should have – I’ve always wanted to do it, so maybe I did look, I can’t remember now but it didn’t faze me […] My plan was always to be a GP and I was quite sad that it didn’t work out that way for me.”* (GP8)
(5) Managing confusion	How the individual seeks out information and knowledge to clarify differences between different specialty career choices, and what this would mean in their individual life	
(5a) Self-awareness	Understanding of self (that is, self-awareness)	*“[…] during medical school and during internship, I didn’t really find one specialty that I was, like, ‘Oh, this is a specialty that I love and I want to do for the rest of my life.’ I found that I did really like that variety and seeing lots of different things.”* (GP1)
(5b) Vocational information: non-GP	Understanding of non-GP specialties as vocations	*“Then after my first year of internship and then I already – it just confirmed what I thought before, which was that I couldn’t see myself working in the hospital. […] the problem is that everyone is really overworked and understaffed pretty much. So it’s not because people don’t want to work. It’s the problem you’re overworked and you don’t have enough resources, enough people. But the problem is everyone needs an extra hand.”* (GP9)
(5c) Vocational information: GP	How general practice, GPs and their values were described and contrasted with other medical norms and non-GP specialties	*“[…] firstly in university, was that I really liked the unit that ran general practice. It seemed to be a unit that actually cared about its students, and it was the first time that I felt that we were able to kind of combine all the medicine that we’ve learnt and really apply in a holistic manner that helped with how we delivered our treatment in the sense of making it patient-centred.”* (GP4)
(5d) Assessing person– environment fit	When the individual is cognitively comparing the person-environment fit for a specialty career choice against their knowledge of the different choices available	*“I wanted the diversity of location, as well. So, I wanted to be able to potentially work in an urban environment or a remote environment, or a regional town or whatever. I guess I felt like I wanted to be able to have the flexibility to do that. Again, I think general practice offers that where a lot of other specialties don’t.”* (GP2)
*Educational learning construct*		
Threshold concepts	Knowledge and experiences that move the individual from confusion (that is, when they don’t know enough about their potential choices) to ambiguity (that is, they know all they can know about their potential choices other than what happens when they step into it themselves) about their GP career choice	*“[…] he was not only working as a GP, but he was also working as like the obstetrician at the rural hospital and working as the anaesthetist at the rural hospital. So he had lots of lots of different hats. So I guess it sort of showed that a GP doesn’t always have to just be like the GP. Like if you do rural training you can do lots of different things. But even in, I guess metro areas, GPs are doing lots of different roles.”* (GP1)

Overall themes for GP pathways were then developed inductively using a reflexive thematic analysis approach^31^ to understand key factors beyond the DTC framework.

### Trustworthiness

Several strategies were used to ensure the trustworthiness of this research. To aid ‘sense-making’^32^ of emergent findings, FY (lead analyst) sought continual feedback from GP researchers in the team and regular in-person visits to the university’s GP academic unit. This included presentation of preliminary findings (FY, MM) for feedback across four workshops and seminars with non-project attendees. Dependability was addressed by engaging in regular discussions and coding. Confirmability required researcher reflexivity throughout the study, wherein the interprofessional team members’ different perspectives, assumptions, biases, and research paradigms were acknowledged and discussed.^33,34^

## Results

Twenty-five interviews of 30–60 minutes were conducted between August and September 2023. Participant demographics are summarised in Table 2. Most were aged 30–39 years, with a relatively even mix of genders. Over half the participants had completed their GP fellowship training between 2021 and 2023 and had graduated from their primary medical degree between 2015 and 2017. Nine participants affirmed their transcripts, with three providing additional clarifications.

**Table 2.** Participating GPs’ demographic summary

Characteristic	Frequency
**Gender**	
Male	10
Female	14
Non-binary/third gender	1
**Age, years**	
<30	2
30-34	15
35-39	8
**Affiliation**	
ACRRM	1
RACGP	23
Both	1
**Rural self-identification (multiple allowed)**	
Rural background	6
Rural medical school term>12 months	7
Rural postgraduate doctor placement>12 months	10
GP training – mostly rural/regional	12
GPs reporting no substantial medical training rural experiences	13
**Number of GP jobs held**	
0	1
1	10
2	7
3	6
4	1
**Main GP job location**	
New South Wales	6
Northern Territory	1
Queensland	13
South Australia	1
Victoria	1
Western Australia	2
Metropolitan (MMM1)	16
Regional centre (MMM2)	3
Small rural town (MMM5)	4
Very remote (MMM7)	1

ACRRM = Australian College of Rural and Remote Medicine. MMM = Modified Monash Model (classification). RACGP = Royal Australian College of General Practitioners.

Narratives of GP career pathways were not typically structured chronologically, but were instead described as influenced by various experiences over time:

*“It [specialty choice] ended up being a bit of a process of elimination. I think everybody kind of goes through this.”* (GP20)

### GP specialty decision-making

Four themes were developed from the interview data to understand participant perspectives on GP specialty decision-making, namely: current gaps in GP career information; person-specialty fit in GP careers; overcoming the influence of medical norms; and positive GP experiences affecting GP career choices.

#### Gaps in GP career information

Most participants did not gain enough direct experience or knowledge about the GP specialty from their medical training. Three overall reasons were outlined: insufficient socialisation of medical students and postgraduate doctors into the GP context; constantly changing governance of GP training pathways; and information about GP careers being difficult to access.

Insufficient socialisation refers to the lack of familiarisation and exposure in GP contexts. Most participants realised they had been unprepared for choosing a GP career. Pre-vocational periods in hospitals seemed to focus understanding on non-GP specialty training, creating comparative disadvantages for choosing a GP career:

*“I think being in the hospital system for the first few years of your training really blocks out that view of general practice…”* (GP17)

After being entrenched in non-GP specialty hospital work during their pre-vocational years, many had chosen GP training to leave hospital working conditions, rather than choosing GP training on merit alone:

*“If you want the actual initial line of reasoning it was […] more opting out of the other [physician training] stuff and then slowly discovering how GP is better, as opposed to the other way around.”* (GP10)

Without widespread postgraduate doctor experience of GP work, GP administration, GP business models, and managing business aspects of private GP work such as fee setting were thus unexpected. This caused alarm for participants when GP training was nevertheless chosen:

*“The recruitment is ‘This is amazing, this is general practice.’ That’s fine. Nowhere in it was there any business training at all. Like you come out [of pre-vocational training] almost not ready.”* (GP3)

Similarly, many felt underprepared in their proficiency and self-efficacy for consultation, diagnosis, treatment planning, and optimising fee setting when running independent GP consultations:

*“…all of a sudden you have to make your own decisions, form a plan, within the scope of 15minutes – [it] was very different to how you operated in the hospital.”* (GP17)

#### Person–specialty fit in GP careers

Multiple, layered reasons were provided for choosing a GP career, including personal goals and lifestyle fit (see Table 3).

**Table 3.** Reasons provided for GP or rural generalist career choices

Reasons (*multiple usually present*)	Timing (context)	Doctor’s characteristics (context)	Exposure (context)
**Environmental**			
Work experience in GP setting	Medical school or pre-vocational, reinforced over time	Open to all specialties, haven’t decided on specific specialties	Generally positive or neutral clinical experience/s in general practice placement, feeling connection or ‘clicking’ with holistic and individualised person-centred care, and/or a patient demographic, and/or a medical community, and/or rural areas, and/or exemplary generalist clinicians
Ruling specialties and medical interests in or out	Medical school and pre-vocational experiences	Open to all specialties, but is becoming more self-aware of own interests, skills, and capabilities over time – discovers they enjoy a broad variety of medicine and work	Experiencing a broad variety of areas of clinical medicine, to the point where they could not choose only one specific specialty to continue in. An ‘aha’ moment often came, realising how different medical areas can be included in GP specialty (with or without training), and GP career could be tailored individually to self
Validation and support experienced in the general practice environment	Medical school and early pre-vocational	Feels vulnerable with lack of skills or knowledge, and is within identity formation as doctor or GP	Medical experiences that were more than observation in the general practice setting with GPs and allied health, ‘feeling helpful’ in the general practice setting compared to other specialty placement experiences, and connecting with GP role models who invested in personal relationships and demonstrated in-depth generalism and continuity of care medicine, while showing that their lifestyles allowed family and other interests to be prominent
Hospital non-GP specialties perceived as less favourable environment to the individual	Pre-vocational	Tired, burnt out, frustrated, may have completed 10+ years of university and hospital vocational training by this stage	Some exposure to general practice setting Usually this mechanism was triggered during pre-vocational training which takes place in hospital settings Hospital administration (which was difficult to appeal to for personal leave and considerations) particularly prompted this conclusion
**Professional**			
GP medical work fits their personal medical interests, after comparing GP and hospital non-GP specialties	Medical school and pre-vocational	Individual prefers to ‘know’ patients over a long period of time through interactional and holistic person-centred care; likes a broad variety of presentations and lesser patient acuity	Exploring different specialties’ skills, attributes, personalities, lifestyle, and own medical interests, preferred values and lifestyle through placements and postgraduate doctor training, including the GP specialty
GP work setting fits their personality, skills and norms, after comparing GP and hospital non-GP specialties	Medical school and pre-vocational	Individual wants to privilege family, personal wellbeing, and external goals, not just medical work	Observations of specialty trainees and consultants, finding out information from trainees and consultants, and personal experience working within different specialties, including the GP specialty
Holds a health system perspective, rather than only privileging their specialty or discipline	Registrar and fellow	The doctor feels quietly providing value to the healthcare system is important through disease prevention interventions and longitudinal episodic care, rather than easily measurable once-off acute hospital care	Experiencing GP work over more than 12 months and realising the value and impact that general practice has on individuals and at a system level
Medical status perceived to matter less than personal rewards derived from helping others	Medical student to fellow	The doctor values being a part of patients’ health progress and lives, enjoys helping others	Experiencing GP work in one practice for more than 12 months, having their own patient pool. Talking about medical status with people external to medical profession.
Registrar training takes less time and requirements than other specialties		The doctor prefers above factors over other specialties’ perceived benefits and long training programmes; wants to plan life outside medical career within 2–5 years rather than 5–10 years	Comparison of non-GP and GP specialty training requirements Speaking with non-GP and GP registrars and consultants
Previous medical experience and credentials (including those gained when pursuing other training pathways) are valued and not ‘wasted’ in GP work and can be used still	Pre-vocational to fellow	The doctor previously began training in or has serious interest in other specialist medical pathways (for example, paediatrics or women’s health) but is interested in pivoting to GP because of incompatibilities in lifestyle, or personal or family goals, and similar reasons	Explanation by senior doctors that other medical experience and credentials are beneficial, welcomed, and acknowledged in GP or rural generalist settings
**Non-professional**			
Work–life balance and personal sustainability is attainable in GP career	Medical school to fellow	Does not believe medical career should ‘take over’ their lives, believes in the importance of self-care and wellbeing, and is willing to take actions to perform self-care	Seeing and knowing GP role models who have built successful and sustainable careers that allow them to be excellent doctors, but also spend time with their families and achieve other goals external to medical career
Agreeable or tolerable projected economic and social position as GP	Registrar, fellow	No financial responsibilities (for example, mortgage, family) undertaken during pre-vocational years, willingness to change medical perspectives, considers GP pay sufficient since they have enough patients and Medicare billing competence/understanding	Comparison with GP pay and status with non-medical careers, rather than non-specialist careers Availability of locum GP and emergency department positions as second job for GP registrar period Understanding and experience in Medicare billing

As noted earlier, many demurred from ‘hospital’ specialties as a result of perceptions of rigid and personally destabilising working environments, perceived strain, lack of work–life balance, and job shortages in planned future living locations:

*“But then once I started working as a doctor in my intern year, I just saw how inflexible training was in other specialties that are hospital-based. Also, the shift work and working on public holidays and all of that – the lifestyle and the quality of life of hospital-based training. So that really led me to general practice.”* (GP1)

Several participants commented positively on how a variety of medical experiences helped to clarify their own interests and fit with the GP specialty. Also, those participants who took a break in their training (for example, to ‘locum’ work across Australia or internationally) seemed to feel more GP career fit: they positively promoted GP careers and appeared more deliberate in their career choices.

To resist dissatisfaction, participants spoke of the necessity of ‘making’ the GP career fit themselves:

*“…there’s a difference between attracting people to general practice and potentially keeping them in general practice. […] I think it’s learning to appreciate what you have an interest in, and then tailoring your GP career towards that, and you can change. […] Because the other side is, if you don’t do general practice that you enjoy, it’s just another dull job.”* (GP13)

For many, this meant exploration for work variety, engagement and flexibility through part-time GP positions plus other roles and qualifications and further study (for example, medical education, university involvement, research, indigenous healthcare or other special interests):

*“I would say there’s more part-time GPs then there are full-time GPs. […] I feel like it’s more of an incentive that you can find other roles […] there’s actually dedicated time to pursue dedicated interests, which I think is a big bonus.”* (GP4)

#### Overcoming the influence of medical norms

Medical profession and GP specialty normative perceptions (that is, norms) seemed to create specific GP career motivators (that is, types of ‘medicine’ practised and individual compatibility) and detractors (that is, perceived lower GP specialty status).

##### GP norm motivators

Individual preferences for types of medicine (see Table 3) were described as an important rationale for choice of medical specialty:

*”[…] general practice was the thing that I always expected to do because of the connection with patients, I guess, and the community-based nature of it.”* (GP2)

*“[…] for myself, I think I’ve got such a good variety in the day. I get to see a bit of everything… I think it just keeps the day interesting and I’m not weighed down by one particular specialty.”* (GP17)

GP-specific medicine preferences included: continuity of care, variety of medical presentations, individualising medical treatment, feeling rewarded by longitudinal patient care and progress, preventative healthcare, and being embedded in the community. GP-specific work preferences included the freedom to choose patient cases or a ‘special interest’ (that is, the ability to ‘be anything you want to be as a GP’), relative clinical autonomy (rather than team-based, hierarchical reporting), and relative work–life balance with schedule flexibility inherently possible for those working as contractors (rather than as employees).

##### Perceived norm detractors

Participants reported that negative perceptions of the GP specialty were often perpetuated by hospital-based supervisors and colleagues. Some described non-GP specialists influencing high-achieving students with comments that good students would be ‘a waste’ in general practice:

*“You have this entire subculture of people who never engage with general practice and who only ever see the worst of general practice and they spend their entire time working with the worst outcomes of general practice […]”* (GP6)

Participants felt such judgments were unfair and influenced medical students and postgraduate doctors away from general practice. Choosing to become a GP thus meant participants had to mentally discard these negative opinions of GP careers:

*”Well, actually, like, yeah, the [medical] status isn’t about why we do these things [person-centred care] really. […] the ‘warm and fuzzies’, as I like to say […] I definitely get that from my work clinically.”* (GP25)

### Positive GP experiences

Positive GP experiences, including the influence of pivotal supervisors, colleagues and transitional schemes, could potentially change specialty career choices towards general practice over time:

*“[…] essentially, what I was sold by a GP was that if I do this pathway, I get to do a bit of GP and I get to pick a special skill. […] I thought ‘This is going to work for me.’ So I decided to head down the GP pathway.”* (GP19)

GP supervisors teaching quality patient care were vital for creating positive impressions:

*“[…] the GP, my supervisor […] put me through my paces, but it certainly made me a good clinician […]”* (GP25)

GP experiences and personal connections were reportedly able to buffer exposure to non-GP specialties and negative attitudes towards general practice. Having GP mentors, participating in GP education or networks, previous or ongoing involvement with university GP education units, and longer GP work exposures of 6–12 months (available only in some rural-generalist focused university programmes) seemed to decrease pre-vocational attraction to non-GP specialties.

Transitional schemes between hospital and general practice settings seemed to provide postgraduate doctors with safe, attractive learning positions for trialling GP work while keeping their specialty options open. Such schemes gave two participants the knowledge, support, mentorship, and confidence to enter general practice training, facilitating key preparation for GP registrar work (for example, interactional skills, generalism philosophy, fluency with Medicare billing numbers, and other administrative tasks):

*”I thought it was really nice that they had this kind of dedicated GP stream and all the other residents in there were very like GP minded. […] That was pretty unique and that’s why I sort of jumped on that opportunity and actually moved hospitals for that reason.”* (GP20)

### Use of career counselling framework for analysis

The quotes in Table 1 illustrate the relevance of the DTC framework constructs for GP career decision-making. Specifically, participants’ confusion about possible specialty careers was managed by experiencing different specialties to understand their individual preferences, skills and goals within the context of community-based (for example, general practice) or hospital-based work. Participants described making anchor choices, such as medical work preferences and participating in opportunities or scholarships within medical training (including moving cities or towns):

*”[…] if you wanted to encourage more people to do it [general practice training], what are people driven by? They’re driven by money, family, relationships, interests.”* (GP3)

Consequential reflective agentic adjustment followed (for example, deciding to pursue GP pathways rather than surgical pathways), affecting subsequent anchor choices. However, ambiguity management was less apparent in their narratives. GP pathway threshold concepts are listed in Supplementary Table 1.

## Discussion

### Summary

This study explored the perceptions of recent GP fellows about their journeys into a general practice career in Australia,^35^ confirming multifactorial decision-making complexity^18,36^ Current training pathways insufficiently address information gaps about GP careers, including GP work structures and related implications on medical care, business aspects of general practice and associated administrative work, and the need to develop independent clinical reasoning and autonomy competencies earlier than those in non-GP hospital careers. Choosing general practice training may thus be a less-informed decision than previously understood.

### Comparison with existing literature

Our findings confirmed that medical norm detractors towards GP careers may be commonly encountered within medical education, as supported by the international literature.^37–41^ Exposure to medical norm GP career motivators seemed comparatively lacking. Personal experiences of positive GP work with sufficient duration to experience key characteristic rewards of GP work such as continuity of care were uncommon, depending largely on universities’ medical school programmes and fortuitous opportunities. Perceptions of less attractive working conditions in hospital specialty training pathways were said to trigger a positive attitude towards GP careers during pre-vocational years. However, the rarity of socialisation into general practice work (particularly administrative and business-related competencies) during medical training years^42^ was reported to negatively disadvantage and burden affected GP trainees. There is some evidence that positive student experiences of GP careers could increase GP career intentions,^39,43–46^ although the effect may be time dependent and fade with further exposure to hospital training.^47^ This imbalance of exposure to insufficient positive motivators and surplus negative detractors of GP careers seems directly related to a dearth of systemised exposure to general practice throughout medical training, which disadvantages integration between healthcare sectors, while potentially creating an ‘othering’ of the GP specialty within medicine.^48,49^

After GP fellowship, participants described continually revisiting career decision making, reinforcing the continuum of GP recruitment and retention which affects training uptake.^50,51^ Understanding underlying rationales and context for choosing and remaining in GP careers, as described in Table 3, may help educators and policymakers to strengthen GP pathways and GP career choice. Participants provided multiple environmental, professional and non-professional reasons for choosing GP careers, tabulated per context (that is, timing, doctor’s characteristics and training exposure required). One of these was enabling GP portfolio careers (that is, being able to hold multiple positions or roles rather than one full-time GP job). Similarly to other studies,^52–56^ many participants had more than one job, demonstrating agentic adjustment to ensure financial, career ambitions, and work variety fulfillment, which were spoken of as helping them remain in general practice. While this took some of their time away from the clinical practice they trained for, a more balanced and varied approach to their work role was reported to be more sustainable. It seems promoting and facilitating this option may be attractive to some postgraduate doctors, despite the loss of GP work hours to activities other than direct care. Given the critical role of GPs in the health system, continued systemic support and reform for a larger and sustainable GP workforce may be necessary, therefore;^57^ retention of current GPs is just as important as postgraduate doctor recruitment and training. Initiatives should span all stages of medical training (for example, university students, postgraduate doctors) but should also include the current GP workforce, who have been experiencing well-documented pandemic burnout.^58,59^ Conversely, rather than expecting sufficient numbers of GP workforce to be solved by the medical profession, we suggest that incentives and sustained initiatives for servicing population health needs should be driven by ‘top-down’ change, for example, focused and long-term collaborations with local primary health networks, and state and federal governments.

The importance of intra-profession narratives has previously been reported as a major challenge to increasing the GP workforce,^38^ suggesting that systemic review and change in the profession’s own maintenance of internal narratives about the GP specialty is required.^60^ Requirements to overcome prevailing perceptions of GP work being of lower status and quality forms a formidable barrier for impressionable postgraduate doctors, particularly when given by respected medical supervisors and lecturers. Such narratives hurt GP pathways and need careful intervention and correction by the whole medical profession, rather than GPs alone.

Congruent with our participants’ report that GP career decisions were a process rather than an event, Xu’s DTC framework and process-focused constructs were highly descriptive of the overall narratives provided. Broad medical career exploration (stage 1) generally occurred within medical education and pre-vocational stages, person–specialty fit information was gathered (stage 2) predominantly during pre-vocational years (with some ambiguity management), and tentative specialty choices (stage 3) were generally re-evaluated (stage 4) during pre-vocational specialty terms. Furthermore, re-evaluation of GP career choices (stage 4) was also evident throughout GP training and fellowship narratives.^29^ Our findings suggest that the approaches to managing ambiguity were less useful, but this may relate to the retrospective nature of our interviews. This study thus provides some evidence for the usefulness of career counselling constructs and the DTC framework^29^ in qualitative research, and specialty decision making within medicine.

Most challenges discussed in our study are not new in Australia nor internationally.^61,62^ While better information about GP pathways and careers should be available throughout medical training for higher uptake, stronger social accountability measures could also be implemented in universities and hospitals to ensure population health needs are addressed.^63^

### Strengths and limitations

This study demonstrates a rich complexity in the individual narratives of GPs who had recently completed all training and addresses a gap in current understanding about GP career decision making in Australia. Our rigorous data collection and analysis processes were conducted by a well-rounded research team with combined expertise and experience in general practice, clinical settings, medical education, and qualitative and participatory action research. Several trustworthiness processes were also completed to ensure rigour. We used a theoretical framework from the career counselling discipline to align our research closely with available evidence, while also advancing the field. However, we note that not all interviewees may have had time to provide further feedback to their interview transcript; some were present in subsequent presentations of preliminary findings and were able to provide feedback then. A key limitation may be the context-specific differences between countries. In particular, globally, many follow an American or Canadian system where medical specialty choices are predominantly completed within medical school, and our findings may not be as salient in those countries, despite the similarities of insufficient graduates choosing family medicine.

### Implications for practice

GP narratives of their specialty career decision making were complex. Understanding their individual person–specialty fit (including preferences and skills, the breadth, variety, and flexibility of a GP career, and the philosophy of generalism towards community continuity of care) was best triggered by several high quality longitudinal experiences in general practice. Many GP choices may not be well-informed decisions because of a lack of information in current medical training, requiring further stakeholder action and policy to address the current GP workforce shortage. Ensuring informed choice of GP training throughout university medical education and hospital pre-vocational training is required, as is counteracting dominant medical narratives about perceived lower GP specialty status. This remains a ‘wicked’ (complex societal) problem that has continued to challenge medicine globally. Findings from this study suggest that a profession-wide and supportive policy approach is required to increase the uptake of GP training to service increasing population health needs for community-based primary care.

## Data

The dataset relied on in this article is available from the corresponding author on reasonable request.
